# Targeting Histone Deacetylases with Natural and Synthetic Agents: An Emerging Anticancer Strategy

**DOI:** 10.3390/nu10060731

**Published:** 2018-06-06

**Authors:** Amit Kumar Singh, Anupam Bishayee, Abhay K. Pandey

**Affiliations:** 1 Department of Biochemistry, University of Allahabad, Allahabad 211 002, Uttar Pradesh, India; amitfbs21@gmail.com; 2 Department of Pharmaceutical Sciences, College of Pharmacy, Larkin University, Miami, FL 33169, USA; abishayee@ULarkin.org or abishayee@gmail.com

**Keywords:** cancer, histone deacetylases, histone deacetylase inhibitors, vorinostat, natural HDACi, apoptosis

## Abstract

Cancer initiation and progression are the result of genetic and/or epigenetic alterations. Acetylation-mediated histone/non-histone protein modification plays an important role in the epigenetic regulation of gene expression. Histone modification is controlled by the balance between histone acetyltransferase and (HAT) and histone deacetylase (HDAC) enzymes. Imbalance between the activities of these two enzymes is associated with various forms of cancer. Histone deacetylase inhibitors (HDACi) regulate the activity of HDACs and are being used in cancer treatment either alone or in combination with other chemotherapeutic drugs/radiotherapy. The Food and Drug Administration (FDA) has already approved four compounds, namely vorinostat, romidepsin, belinostat, and panobinostat, as HDACi for the treatment of cancer. Several other HDACi of natural and synthetic origin are under clinical trial for the evaluation of efficiency and side-effects. Natural compounds of plant, fungus, and actinomycetes origin, such as phenolics, polyketides, tetrapeptide, terpenoids, alkaloids, and hydoxamic acid, have been reported to show potential HDAC-inhibitory activity. Several HDACi of natural and dietary origin are butein, protocatechuic aldehyde, kaempferol (grapes, green tea, tomatoes, potatoes, and onions), resveratrol (grapes, red wine, blueberries and peanuts), sinapinic acid (wine and vinegar), diallyl disulfide (garlic), and zerumbone (ginger). HDACi exhibit their antitumor effect by the activation of cell cycle arrest, induction of apoptosis and autophagy, angiogenesis inhibition, increased reactive oxygen species generation causing oxidative stress, and mitotic cell death in cancer cells. This review summarizes the HDACs classification, their aberrant expression in cancerous tissue, structures, sources, and the anticancer mechanisms of HDACi, as well as HDACi that are either FDA-approved or under clinical trials.

## 1. Introduction

Cancer is the second leading cause of death worldwide and caused 8.8 million deaths in 2015. Globally, 1 out of 6 deaths is because of cancer. Low and middle-income countries are the hotspot of cancer deaths, accounting for approximately 70% of deaths. Lung cancer is the most common cause of cancer death worldwide. According to the National Center for Health Statistics about 1.73 million new cancer cases and 0.6 million cancer deaths are projected to occur in the United States in 2018 [1]. Cancer results from altered cell physiology leading to self-sufficient growth potential, loss of cell cycle control, extended angiogenesis, delay in replicative senescence, dysregulated apoptosis, invasion, and metastasis [2,3]. Progression of the disease is not only governed by genomic and genetic changes, such as translocation, amplification, deletion and point mutation, it also involves epigenetic changes; i.e., alteration in the pattern of gene expression without changing underlying DNA sequence. Methylation of DNA, histone protein modifications and non-coding RNA-mediated gene silencing are the major epigenetic changes, reversible in nature [4]. 

Chromatin is a compact and highly ordered structure comprised of DNA and histone protein. Nucleosome, the basic unit of chromatin, is made up of 147 bp of DNA superhelix wrapped around histone core protein containing two copies each of H2A, H2B, H3 and H4. H1 is the linker histone. The core plays an important role in establishing interactions between the nucleosomes and within the nucleosome particle itself. N-terminals (histone tails) of core histones are flexible and unstructured while the rest of histone proteins are basically globular and highly ordered. Depending on the epigenetic changes in histone tail, chromatin undergoes various conformational changes responsible for upregulation or downregulation of respective genes [5,6]. The common posttranslational modifications occurring in histones are acetylation, methylation, phosphorylation, sumoylation, ubiquitinylation, and ADP ribosylation.

**Figure 1:**
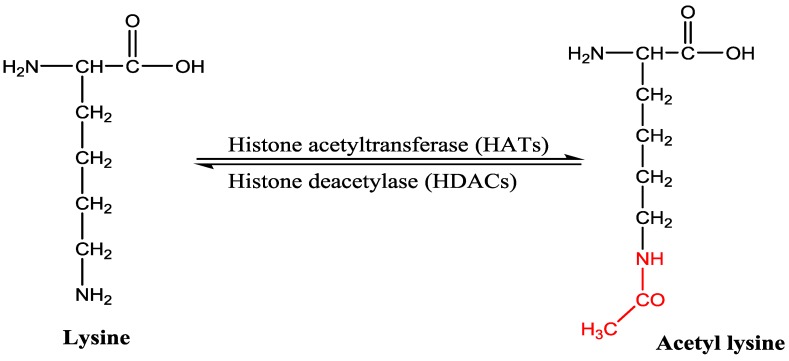
Histone acetylation at the N-terminus lysine by histone acetyltransferases (HATs) and deacetylation by histone deacetylases (HDACs).

Acetylation of a lysine residue of histone was discovered by Vincent Allfrey and colleagues in 1964 and based on the finding it has been proposed that acetylation of ε-amino group of lysine residues could play a role in gene expression [7,8]. Acetylation and deacetylation of N-terminal ε-amino group of lysine residues are regulated by two enzymes, namely histone acetyltransferase (HAT) and histone deacetylase (HDAC) (Figure 1). Acetylation neutralizes the positive charge and decreases the affinity between the histone and DNA helix responsible for relaxation of conformation and greater accessibility to transcription machinery [4,9]. Therefore, acetylation is generally associated with gene activation, however, deacetylation catalyzed by HDAC induces chromatin condensation and downregulation of gene expression. N-terminal acetylation of lysine residue also occurs in non-histone proteins, such as cytoplasmic proteins, transcription factors responsible for alteration in gene expression and other cellular processes [10].

Imbalance in the activities of enzymes HATs and HDACs is responsible for the development and progression of wide variety of cancers [2]. Histone deacetylase inhibitors (HDACi) increase the level of acetylated lysine residues of core histone which in turn restarts the expression of silenced regulatory genes in the cancerous cell and therefore, HDACi are now emerging as anticancer agents [11].

Epidemiological studies have suggested that vegetables, fruits, whole grains, microorganism-derived bioactive components, and fatty acids provide protection against some forms of cancer and other diseases without detectable side effects [12,13,14]. Many dietary compounds that have been identified as having HDAC-inhibitory activities implicated in therapeutic potential in the context of a whole food [11,15]. In the present review an effort has been made to highlight the role of HDACs in the tumor initiation and progression and their inhibitors from natural (dietary and non-dietary) as well as synthetic sources in the management of cancer either alone or in combination with other chemotherapeutic drugs/radiotherapy. 

## 2. Classification of HDACs 

**Figure 2:**
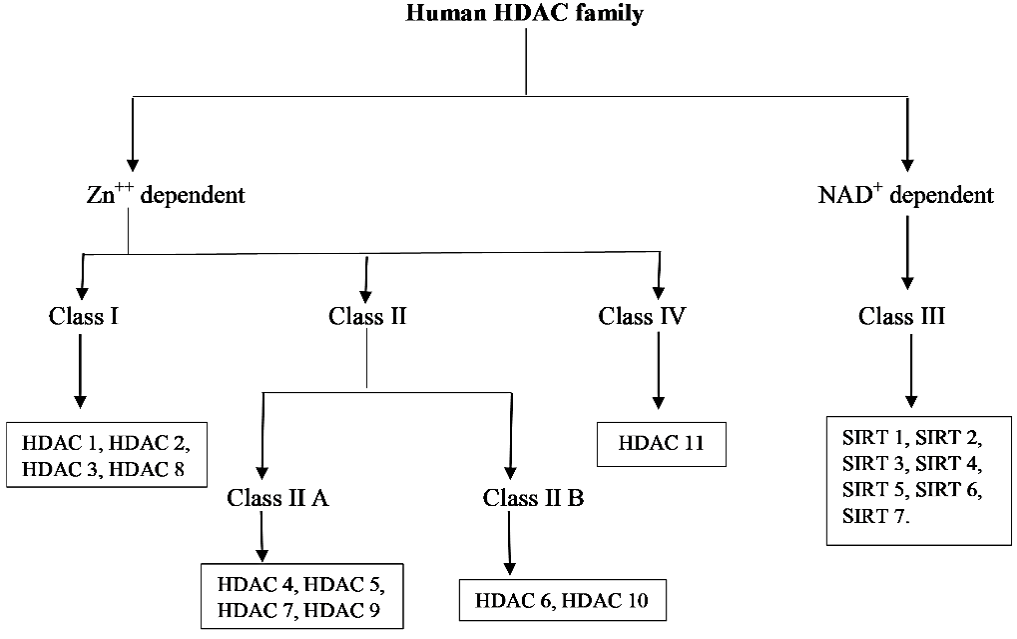
Classification of HDAC family.

In humans, 18 HDACs have been identified so far and are divided into two families and four classes based on their sequence homology to *Saccharomyces cerevisiae* HDACs (Figure 2) [16]. One of the family group members are zinc-dependent, they require Zn^++^ as a cofactor for their deacetylase activity and include HDAC 1 to HDAC 11. HDACs 1, 2, 3 and 8 are grouped into class 1 having a sequence similarity with yeast reduced potassium dependency-3 (Rpd3) and class II HDACs are subdivided into class IIA and Class IIB that include HDACs 4, 5, 6, 7, 9 and 10 which are reported to have sequence homology with yeast histone deacetylase-1 (hda-1) while HDAC 11 of class IV share sequence similarity with both classes of yeast deacetylase Rpd3 and hda-1. 

Another group of the family requires nicotinamide adenine dinucleotide (NAD^+^) as a cofactor for deacetylase activity classified as class III, has sequence similarity to yeast deacetylase silent information regulator-2 (Sir2) and includes seven members from sirtuins (SIRTs) 1 to 7. Sirtuins are known to regulate several cellular processes; e.g., survival, aging, stress response, and various metabolic processes. The members of class I and IV are located in the nucleus while class IIA is mainly located into the cytoplasm and class IIB is found shuttling between the nucleus and cytoplasm. Cellular localization of class III HDACs are nucleus, cytoplasm, and mitochondria [11,17]. Nomenclature of class I, II and IV HDACs are based on their chronological order of discovery; for example, both HDAC 1 and 2 were discovered in 1996 while HDAC 2 was discovered a few months after HDAC 1 [18,19]. Later on, HDAC 3 was discovered in the subsequent years [20]. While HDACs 4, 5, and 6 were first reported in 1999, the HDAC 7 was discovered in early 2000 and so on [21,22]. Table 1 summarizes the HDACs classification, number of amino acids, cellular and chromosomal locations, biological functions, relevant histone/non-histone target proteins, and their expression pattern [6,23,24]. 

## 3. Cellular Targets of Histone/Non-Histone Protein Acetylation

The acetylation or deacetylation status of histone proteins and transcription factors modulate the gene expression pattern. Hyperacetylation of lysine residues of histone proteins promotes the relaxed state of chromatin and activates gene expression [25]. Besides this, acetylation of transcription factors affects their cellular localization. For example, signal transducer and activator of transcription 1 (STAT 1) and nuclear factor-κB (NF-κB) are internalized into the nucleus from cytosol after the acetylation of specific lysine residues where they activate transcription of respective genes. The activity of other transcription factors, such as p53 and FOXO, is also positively regulated by the acetylation process. Acetylation process also affects the stability of proteins, i.e., acetylation of p53, p73, and mothers against decapentaplegic homolog 7 (SMAD 7) prevents their ubiquitinylation and degradation. Interestingly, cell mobility is also affected by the acetylation pattern of α-tubulin and cortactin. It has been reported that HDAC 6 and SIRT2 cause deacetylation of α-tubulin, which promotes microtubule depolymerization and therefore increases microtubule dynamics and cell mobility. Acetylation also affects the activity of retinoblastoma protein (pRB) by blocking its cyclin E-cdk2 dependent phosphorylation so acetylation-dependent hypophosphorylation causes cell cycle arrest [26,27].

## 4. HDAC Mutations in Cancer

Mutations of HDACs have also been observed. HDAC2 mutation in human epithelial cancer resulted in microsatellite instability. Interestingly, a truncating mutation of HDAC2 in human cancers confers resistance to HDACi. These findings suggest that the HDAC2 mutational status of patients should be assessed before therapies using HDACi [28]. HDAC3 are associated with DNA damage control response. Inactivation of HDAC3 causes genomic instability. HDAC4 acts s transcriptional repressor and its mutations have been identified at significant frequency in breast and colorectal cancers. SIRT 2 acts as a tumor suppressor and mutation in its catalytic domain eliminates its enzymatic activity, which compromises the mitotic checkpoint, contributing to genomic instability and tumorigenesis. HDAC9 and 10 are reported to be involved in homologous recombination, and depletion in HDAC 9 and 10 resulted in inhibition of homologous recombination [11].

## 5. HDACs and Cancer: Expression Pattern and Function 

Altered acetylation level and mutation/or aberrant expression of various HDACs have been observed frequently in numerous human diseases including cancer, hence making them an important drug target [2]. Fraga et al. (2005) reported that change in genome-wide patterns of acetylation may lead to the initiation and progression of cancer by demonstrating that cancer cells undergo a loss of acetylation at lysine 16 of H4 [29]. HDACs have various histone and non-histone protein targets that not only regulate the chromatin activity, but also control apoptosis, cell cycle progression and differentiation. Association of HDACs with regulatory processes reflects their involvement in cancer phenotypes [30]. 

### 5.1. Class I HDACs 

#### 5.1.1. HDAC 1 

HDAC 1 overexpression has been reported in Hodgkin’s lymphoma (HL), gastric, ovarian, and prostate cancers [11,23]. Choi and co-workers (2001) have shown the overexpression of HDAC 1 in 60% cases compared with normal tissue [31]. This study was further validated by a recent study including 293 gastric cancer samples showing upregulation in expression of HDACs 1, 2, and 3 [32,33]. Elevated level of expression of HDAC 1, 2, and 3 was reported in pancreatic cancer involving 192 samples and this overexpression was responsible for dedifferentiation and enhanced proliferation of pancreatic cancer cell [34]. Overexpression of HDAC 1, 2, and 3 is associated with mortality rate in colorectal cancer. Expression of HDAC 2 has emerged as an independent prognostic marker in colorectal cancer [35]. Furthermore, overexpression of HDAC 1 is reported in hepatocellular carcinoma [36], lung cancer [37] and breast cancer [38]. Direct correlation between HDAC 1, 3 expression and estrogen and progesterone receptor expression have been reported by Krusche et al. [39]. 

HDAC 1 induces cell proliferation and inhibition of differentiation and apoptosis [23,38]. HDAC 1 and 3 knockdowns (KD) resulted in the inhibition of cell proliferation in Hela cells [40]. HDAC 1 KD has been shown to result in cell cycle arrest either at the G1 phase or at the G2/M transition phase, causing loss of mitotic cells, inhibition of cell growth, and an increase in the number of apoptotic cells in osteosarcoma and breast cancer cells. However, HDAC 2 KD showed no such effects in these cells [41]. HDAC1 might also be involved in multidrug resistance as they showed overexpression in a pattern in chemotherapeutically resistant neuroblastoma cells [42].

#### 5.1.2. HDAC 2

Overexpression of HDAC2 reported in uterine, cervical, gastric, cutaneous T cell lymphoma (CTCL), HL, prostate and colorectal cancers [38]. Elevated expression of HDAC 2 along with HDAC 1 and 3 are associated with advance stage of disease and prognosis of gastric, colorectal, and prostate cancers [33,34,35]. 

Knockdown of HDAC2 in cervical cancer causes increased apoptosis and the differentiated phenotype of cells associated with increased p21^Cip1/WAF1^ expression that was independent of p53 [43]. In breast carcinoma cells, HDAC 2 KD results into the increased DNA binding activity of tumor suppressor protein p53, which causes the inhibition of cell proliferation and induction of cellular senescence [44]. HDAC 2 KD also causes decreased viability, growth arrest, and increased apoptosis in colorectal and breast cancer cells [45]. 

#### 5.1.3. HDAC 3

HDAC 3 overexpression has been reported in HL, ovarian and lung cancers, colon cancer, and chronic lymphocyte leukemia (CLL) [11,38,46]. An upregulated expression of HDAC1 along with HDAC3 was paradoxically related to disease-free survival in invasive breast cancer patients [24]. HDAC3 elevated expression together with HDAC 1 and 2 significantly causes poor prognosis in gastric, colon, and prostate cancers [33,34,35]. Decreased expression of HDAC 3 has been observed in liver cancer [45].

HDAC 3 KD in acute promyelocytic leukemia (APL) cells causes restoration of expression of a retinoic acid-dependent gene whose transcription repression was caused by promyelocytic leukemia retinoic acid receptor alpha (PML-RARα) [47]. While HDAC3 KD in colon cancer causes a decrease in the viability of cell by increasing the rate of apoptosis [45].

#### 5.1.4. HDAC 8

Expression of HDAC 8 has been reported to increase in childhood neuroblastoma. HDAC KD shows reduction in proliferation of lung, colon, and cervical cancer. In childhood neuroblastoma cell HDAC 8 KD causes cell cycle arrest, reduction in cell proliferation [48,49]. HDAC 8 has also been reported in controlling telomerase activity [50].

### 5.2. Class IIA HDACs

#### 5.2.1. HDAC4 

A breast cancer sample showed overexpressed genotype of HDAC 4 [51]. However, lung and colon cancer analysis showed downregulated expression of HDAC 4 [11].

In APL, HDAC 4 was found to repress the expression of differentiation-associated genes by interacting with a leukemic fusion protein, PLZF-RARa [52]. HDAC 4 also regulates the activity of hypoxia inducing factor-1α (HIF-1α). HDAC4 has also been shown to help prostate cancer cells overcome hypoxic conditions by stabilizing HIF-1α. The binding of HDAC4 to HIF-1α generates a complex that regulates glycolysis and the cytotoxic stress of cell adaptation to hypoxic conditions [53]. HDAC4 KD in colon and glioblastoma cells causes an increased apoptosis rate and reduced growth rate [54]. 

#### 5.2.2. HDAC 5

HDAC 5 overexpression has been reported in medulloblastoma [55] and colon cancer [51] while interestingly, the lung cancer sample showed a downregulated genotypic expression of HDAC5 [11]. HDAC 5 traverses from the nucleus to the cytosol upon interacting with transcription factor GATA-1 during differentiation of mouse erythroleukemia cells [56]. Knock down of HDAC 5 has been shown to cause reduced growth and viability of medulloblastoma cells [55].

#### 5.2.3. HDAC 7 

They are highly expressed in ALP, CLL, and colon cancer [46,51] and downregulated expression is reported in lung cancer samples [45,57]. HDAC 7 silencing in endothelial cells altered their morphology, their migration, and their capacity to form capillary tube-like structures in vitro but did not affect cell adhesion, proliferation, or apoptosis, suggesting that HDAC7 may represent a rational target for anti-angiogenesis in cancer [58].

#### 5.2.4. HDAC 9 

Elevated expression of HDAC 9 was reported in ALL and medulloblastoma [59,60] and in cervical cancer [31]. Silencing HDAC 9 results into inhibition of homologous recombination, sensitivity towards DNA damage and decreased viability and growth of medulloblastoma [45]. 

### 5.3. Class IIB HDACs

#### 5.3.1. HDAC 6

A significantly higher expression of HDAC 6 was reported in oral squamous cell carcinoma, hepatocellular carcinoma, acute myeloid leukemia, CLL, breast cancer, CTCL and ovarian cancer, whereas its expression increased in the advanced stages of cancer compared to the early stages of cancer [5,61]. 

HDAC 6 overexpression causes increased migration of the fibroblast cell while the inhibition of HDAC 6 results in decreased fibroblast cell migration [62]. HDAC 6 targeted inhibition causes HSP 90 protein acetylation and disruption of its chaperone activity resulting in decreased viability of K562 leukemic cell [63]. HDAC4/6 has the potential to help prostate cancer cells overcome hypoxic conditions by stabilizing HIF-1 α [53]. HDAC 6 is also involved in metastasis, epithelial to mesenchymal transition in lung cancer cells by compromising TGF-β SMAD 3 pathway [64]. 

#### 5.3.2. HDAC 10

Osada and colleague [57] described the potential role of HDAC 10 in cancer initiation and progression and reported the downregulated expression of HDAC 10 in non-small lung carcinoma cells and this is related with poor prognosis in lung cancer patients. Its role in gastric cancer and CLL is also reported [65]. 

HDAC 10 are shown to regulate the production of reactive oxygen species in gastric cancer cells [66]. HDAC 10 KD causes reduced vascular endothelial growth factor of receptor 1 and 2 (VEGF 1 and 2) and increased sensitivity towards DNA damage in cancer cells [67]. 

### 5.4. Class III HDACs

#### Sirtuins

Growing evidence supports the relation between cancer and sirtuins. As other HDACs they are also both tumor suppressors as well as pro-oncogenic in nature. SIRT 1 expression was shown to be upregulated in acute myeloid leukemia (AML), non-melanoma skin cancer and prostate cancer [68,69,70] while downregulated in colorectal cancer [51]. SIRT 2 expression decreased in glioma and gastric cancer [71]. However, a mutation in the catalytic domain of SIRT 2 has also been reported [72]. Expression pattern of SIRT 3 has been found to increase or decrease in different kinds of breast cancer tissues [73]. SIRT 6 expression was reported to decrease in liver cancer while increased expression pattern in CLL [46]. SIRT 7 shows an upregulated expression pattern in breast cancer [73].

SIRT 2 functions as a tumor suppressor protein and the mutation in its catalytic domain results in compromised cell cycle checkpoints leading to genomic instability and tumorigenesis [74]. In many cancerous cells, increased expression of SIRT 1 induces the p-glycoprotein expression responsible for chemotherapeutically resistant cancer cells whereas siRNA-mediated SIRT 1 KD causes a reversal of drug-resistant phenotype [75]. SIRT 3 functions both as a tumor suppressor and a tumor promoter protein. Higher expression of SIRT 3 was responsible for preventing bladder cancer cells from p53-mediated cell growth arrest [76]. SIRT 7 was shown to stabilize cancer cell phenotypes. SIRT 7 KD results in the tumorigenic potential of human cancer xenograft in mice [77].

### 5.5. Class IV HDACs 

#### HDAC 11

The involvement of HDAC 11 in cancer is not well understood. However aberrant expression of HDAC 11 were reported in HL and Philadelphia-negative chronic myeloproliferative neoplasms (CMPN) [78]. 

siRNA-mediated HDAC 11, KD results in an increased apoptosis rate in HL, colon, prostate, breast, and ovarian cell lines [45,79]. Selective inhibition of HDAC 11 with histone deacetylase inhibitor (HDACi) reduces the chance of splenomegaly and other metabolic disorders reported in CMPN affected people [78].

## 6. Histone Deacetylase Inhibitors as an Anticancer Agent

The aberrant or altered expression of HDACs or their functions is frequently observed in a variety of cancer types and is the reason for targeting HDACs in cancer therapy. The availability of HDACi not only has accelerated our understanding of HDAC functions and its mechanism of actions but also presented a promising new class of compounds for cancer treatment. HDACi belong to a large and diverse family of both natural and synthetic compounds and can be categorized into four groups; i.e., hydroximic acid, benzamides, cyclic peptides, and aliphatic fatty acids [80]. Several natural or synthetic compounds are isolated and characterized for their potential as histone deacetylase inhibitors. The first significant compound identified as HDACi was *n*-butyrate, responsible for the accumulation of hyperacetylated histone inside the nucleus [81]. Subsequently, trichostatin A (TSA) and trapoxin A were found to be reversible and irreversible inhibitors of HDACs, respectively [11,82]. Schreiber et al. (1996) were first to discover and clone the human HDAC by using trapoxin A [80]. While developing these compounds as anticancer agents, parameters like their specificity, efficiency, pharmacokinetic and toxicological properties were analyzed [83]. Some HDACi, such as TSA, lycorine and zerumbone, are pan-HDAC inhibitors since they act on all isoform of zinc-dependent HDAC classes, while others are class specific.

HDCAs can reversibly modify the acetylation pattern of histone/non-histone protein resulted in abnormal gene expression without changing the DNA sequence. HDACi can overcome this problem and can resume the expression of tumor the suppressor genes responsible for apoptosis, cell cycle arrest, and the inhibition of angiogenesis and metastasis. Ungerstedt and co-workers [84] reported that cancerous cells are more sensitive than normal cells towards HDACi-induced apoptosis.

### 6.1. Natural HDACi

Altogether natural compounds provide pleiotropic and potent inhibitors of all hallmarks of cancer. Many of the HDACi discovered to date are of natural origin; for example in 1976, Tsuji et al. [85] isolated a naturally occurring HDACi, TSA from *Streptomyces hygroscopicus*. FK322, a cyclic peptide isolated from *Chromobacterium violaceum* selectively inhibits the activity of HDAC 1 and 2. TSA causes differentiation of cell and arrests the cell cycle of both normal and cancerous cells, resulting in the accumulation of acetylated histones [86]. Depudecin and trapoxin A and B are also the examples of naturally occurring HDACi extracted from a fungus. Marine organisms are also the source of natural HDACi, such as largazole and azumamides, and they are reported to be active even at nanomolar concentrations [11]. Other well-characterized naturally occurring HDACi, such as butein, kaempferol, protocatechuic aldehyde, sinapinic acid, resveratrol and zerumbone, are isolated from plant, fruits or vegetables (Table 2). Molecular modelling studies revealed the HDACi like activity of other dietary compounds; i.e., vitamin E, α-lipoic acid, and biotin [48]. For the first time, the clinical validation of natural HDACi was done by Riggs and colleague [81] in 1977. They analyzed the effect of butyrate on histone modificationin HeLa and Friend erythroleukemia cell lines [80]. Later in 1980, McKnight et al. [87] reported the effect of propionate on histone deacetylation in chick oviduct and showed it to have lesser activity than butyrate. Both these compounds were active at millimolar concentrations and synthesized by colonic bacteria. Valproic acid, a longer chain aliphatic fatty acid, also reported to have significant HDACi activity. Valproic acid inhibits HDACs activity by binding to its active site [87]. Detailed features of other natural histone deacetylase inhibitors and their sources are shown in Table 2.

### 6.2. FDA-Approved and Under Clinical Trial HDACi

Aberrant expression of HDACs gene is reported in various cancer types. So, the scientists across the globe are searching for a therapeutic alternative, which not only can inhibit the increased activity of HDACs, but also reverse the malignant phenotype. The histone deacetylase inhibitors chiefly cause the gene to be in a hyperacetylated state, which in turn restarts the gene expression, and are also involved in chromatin stability, mitosis, and the DNA repair mechanism [23]. However, it has been reported that both normal and cancerous cells show accumulation of acetylated histone after treatment with HDACi, but healthy cells appear to be much less susceptible to apoptosis and growth inhibition than a cancerous cell. Four synthetic compounds, viz., vorinostat, romidepsin, belinostat, and panobinostat have been approved as HDACi for cancer treatment to date by the United States Food and Drug Administration (FDA). In addition, many other HDACi are under clinical trials in patients suffering from various types of cancer [101,102]. FDA-approved and other HDACi under clinical trials are presented in Table 3.

Vorinostat, a hydroximic acid-based drug, is also known as suberanilohydroxamic acid (SAHA) and is marketed with different names. Zolinza was the first FDA-approved vorinostat drug for cutaneous T cell lymphoma treatment (CTCL). It inhibits the HDACs except class III sirtuins and was developed by Merck & Co. (Kenilworth, NJ, USA). In phase II clinical trial study (NCT00958074) on 74 CTCL patients with a daily oral dose of vorinostat (400 mg), an objective response rate (ORR) of 30% was observed [11]. Vorinostat is also reported to be useful in other cancer types, such as brain metastasis, refractory colorectal, advanced solid tumors, melanoma, pancreatic, lung cancer and multiple myeloma [6]. A combinatorial therapy of vorinostat with temozolomide and radiotherapy is under clinical trial for the treatment of early stage of glioblastoma multiformae (NCT00731731).

Romidepsin, a cyclic tetrapeptide, was the second FDA-approved drug for treatment of CTCL in 2009 and in 2011, for treatment of peripheral T cell lymphoma (PTCL) with the response rate of 34% and 25%, respectively. However, romidepsin treatment was associated with side effects, such as nausea, vomiting, cardiac toxicity, and myelotoxicity [11,37]. Romidepsin has been evaluated for treatment of T-cell lymphoma either as a single agent (NCT00426764) or in combination with other drugs (NCT03141203) in 30 clinical trials. Romidepsin intake produced fatigue, nausea, vomiting, diarrhea, constipation, phlebitis, headache, and dyspnea as side effects [6]. Romidepsin either singly or in combination with paclitaxel exhibited elimination of both primary tumors and a metastatic lesion at multiple sites formed by the SUM149 IBC cell line in the Mary-X preclinical model [103].

In 2014, belinostat was the third drug approved by the FDA for PTCL treatment. It is a hydroxamate that inhibits the activity of class I and II HDACs. Clinical trial study (NCT00865969) on 120 PTCL patients showed an ORR of 26% [6]. Belinostat is now being investigated in more than 15 clinical trials for the treatment of CTCL (NCT00274651), multiple myeloma (NCT00431340), Burkitt lymphoma (NCT00303953), and solid tumors as in fallopian tube cancer (NCT00301756) [23].

Panobinostat was approved by the FDA in 2015 for the treatment of multiple myelomas [37]. It is a hydroxamate derivative causing the inhibition of class I, II, and IV HDACs. An ORR of 27% was reported for panobinostat. Diarrhoea and cardio-toxicity are side effects associated with Panobinostat. It has been used for treating other cancer types, such as CTCL (NCT00490776), AML (NCT01613976), Hodgkin’s lymphoma (NCT00742027), MDS (NCT00594230), thyroid carcinoma (NCT01013597), and colorectal and prostate cancers (NCT00663832) in more than 50 clinical trials [23]. 

In contrast to the above mentioned clinical trials and studies reporting efficacy of HDACi in the treatment of various lymphomas, leukemia and myeloma, solid tumors have shown limited response against HDACi. The study conducted by Paller and colleagues [104] reported that HDACi VPA and vorinostat in combination with AMG 900, a pan-aurora kinase inhibitor, significantly enhanced cellular senescence, polyploidy, and apoptosis in prostate cancer cell lines (DU-145, LNCaP and PC3) as compared with a single agent treatment. Combination therapy with Janus kinase (JAK) inhibitor INCB018424 has been shown to improve the clinical efficacy of vorinostat in triple-negative breast cancer patients [105]. Similarly, combination therapies targeting HDACs and IκB kinase have shown potential against ovarian cancer [106].

## 7. Mechanisms of Action of HDACi

HDACi induces cell cycle arrest, differentiation, and apoptosis as well as inhibits angiogenesis [119]. The mechanism of the anticancer effect of HDACi depends upon the cancer type, individual, stage of cancer, dose, and some other factors [120]. The antiproliferative mechanisms of HDACi action are described below.

### 7.1. Cell Cycle Arrest

Various mechanisms are involved in HDACi-mediated cell cycle arrest. One of the most important mechanisms is the increased expression of the cyclin-dependent kinase (CDK) inhibitor gene CDKN1A (p21, WAF1/CIP1). An interesting fact is that the HDACi-mediated overexpression of p21 is independent of p53 [121,122]. The concentration-dependent cell cycle inhibitory effects of HDACi have been observed. At lower concentrations HDACi predominately induces G1 arrest while at higher concentrations induces both G1 and G2/M arrest [123]. p21 is mainly associated with G1 and G2/M arrest, inhibits the activity of CDKs (i.e., CDK 4/6), which regulates the progression of the G1 stage of cell cycle, CDK 2 responsible for G1/S transition and cdc2/CDK 1 causes G2/M transition. The p21 mutation abolishes the HDACi induced G1 arrest [124]. However, HDACi-mediated G1 arrest is also observed in a cell without p21. Hitomi et al. [125] reported that TSA causes G1 arrest in human colon p21 mutant cell by induction of p15 (INK4b), which subsequently causes inhibition of cyclin D-dependent kinases resulting in the absence of CDK 2. Protein p53 interacts with the p21 promoter by competing with HDAC1 and alters the expression of the p21 gene. HDAC1 is the transcriptional repressor of p21, which gets detached from the Sp1 (promoter-specific RNA polymerase II transcription factor) after HDACi treatment, resulting in increased p21 expression. Moreover, HDACi also causes an increase in the half-life of protein p53 thereby improving its interaction with p21 and increased levels of p21 inside the cell mediates cell cycle arrest and apoptosis [126]. HDACi treatment also compromises the CDK activity, which may account for the dephosphorylation of the retinoblastoma protein (Rb), which blocks the elongation factor, E2F function in the transcription of genes for G1 progression and G1/S transition [118].

### 7.2. Induction of Apoptosis in Transformed Cell

**Figure 3:**
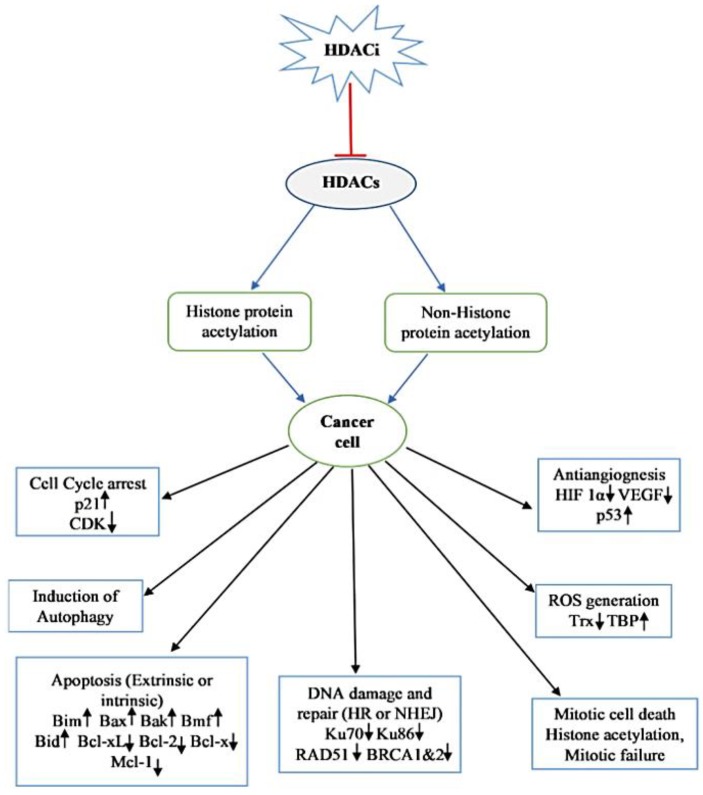
Multiple anti-tumor pathways such as, cell cycle arrest, induction of autophagy and apoptosis, DNA damage repair, ROS generation, angiogenesis inhibitor and mitotic cell death are activated by the action of HDACi in cancer cells. Arrows (↑ and ↓) indicates the increase and decrease, respectively, in the obtained variables. HIF, Hypoxia inducing factor; VEGF, Vascular endothelial growth factor; HR, homologous recombination; NHEJ, Non-homologous end joining; Trx, Thioredoxin; TBP, Thioredoxin binding protein.

HDACi induces the rate of apoptosis in a transformed cell by regulating both pro-apoptotic and anti-apoptotic genes (Figure 3) and this involves the activation of both extrinsic and intrinsic apoptotic pathways. HDACi induced extrinsic pathway initiation involves the binding of death receptor such as Fas (Apo-1 or CD95), tumor necrosis factor (TNF) receptor-1 (TNFR-1), TNF-related apoptosis-inducing ligand (TRAIL) receptors (DR-4 and DR-5), DR-3 (Apo3) and DR-6 to their ligand FasL, TNF, TRAIL, and TL1A resulting into activation of caspase 8 and 10. In vitro and in vivo studies suggest that HDACi upregulates the expression of both death receptor and their ligand in transformed cells but no such effect is observed in normal cells [127]. Upregulated expression of Fas and FasL have been reported in the treatment of human neuroblastoma cells with m-carboxycinnamic acid bihydroxamide, nude mice xenograft of osteocarcinoma with FK228, acute promyelocytic leukemia model of a rat with valproic acid, and so on [128,129]. FK228 also induces the expression of TNF α in HL 60 and K562 cells. cFLIP, an inhibitor of the activity of the death receptor has been reported to be downregulated after HDACi treatment in cancer cells [127]. HDACi-mediated apoptosis also involves the activation of the intrinsic apoptotic pathway, which causes the release of inter mitochondrial membrane protein, such as cytochrome c, Smac, apoptosis-inducing factor (AIF), and the subsequent activation of caspase. HDACi activate the intrinsic apoptotic pathways by regulating the transcription of pro-apoptotic genes; i.e., Bid (BH3 interacting domain death agonist protein), Bad (Bcl-2 associated agonist of cell death protein), and Bim [130]. The mechanism of HDACi-mediated intrinsic pathway activation is not well understood, but it is suggested that HDACi causes leaking of mitochondrial intermembrane proteins, cytochrome c, AIF and Smac. The release of cytochrome c from mitochondria in turn causes activation of caspase-9 [131,132]. The intrinsic apoptotic pathway is regulated by changing the HDACi-mediated expression of factors. It can be concluded that HDACi upregulate the expression of proapoptotic genes and pathways (BAX, Apaf1 and BAK) and downregulate the expression of the antiapoptotic Bcl-2 family proteins, Bcl-2, Bcl-XL and Mcl-1 [133]. 

### 7.3. Autophagic Cell Death

The role of HDACi in autophagy is not well understood. Recent findings suggest that autophagy serves as cell death mechanism, therefore, autophagy inhibitors or knockdown of autophagy-related gene decreases the anticancer effect of HDACi. When HeLa cell line having an Apaf-1 knockout or Bcl-XL overexpression was cultured with vorinostat or butyrate, it undergoes autophagic cell death by forming autophagosome inside cytoplasm [134]. Treatment of a colon carcinoma cell HCT116 with vorinostat resulted in the inhibition of cell growth as well as senescence type phenotype [135]. SAHA causes cell death in endometrial stromal sarcoma cell via autophagy [136]. In p53 mutant cancer cells, SAHA induces autophagy and cell death. HDACi-mediated autophagy involves several signaling pathways, one such pathway is the mechanistic target of the rapamycin (mTOR) pathway that is the main suppressor of autophagy via phosphorylation and the inactivation of the Unc51 like autophagy activating kinase 1 (ULK1) complex. SAHA inhibits the activity of mTOR and resumes the activity of ULK1, upstream component of autophagy pathway [137]. SAHA up-regulates the expression of autophagy-related protein by stimulating NF-κβ activity [138]. SAHA also induces autophagy via ROS production in leukemic and hepatocellular carcinoma cells. Romidepsin induces autophagy in HeLa cells [139]. SAHA inhibited the growth of glioblastoma cells xenograft in nude mice by inducing autophagy via downregulation of AKT-mTOR signaling [140]. In some cancer cells showing resistance towards apoptosis, HDACi can induce cell death via the induction of autophagy by bypassing the apoptosis. Hence, HDACi induced autophagy stimulation could be a promising anticancer strategy.

### 7.4. Inhibition of Angiogenesis

HDACi mediated inhibition of angiogenesis can interfere with the metastasis. HDAC inhibitors downregulate proangiogenic genes, such as the vascular endothelial growth factor gene (VEGF) and endothelial nitric oxide synthase gene [141]. The inhibition of the activity of hypoxia-inducible factor (HIF) by HDACi can block the angiogenesis. Hypoxia is a commonly occurring condition in tumor cells responsible for the overexpression of class I HDACs, except HDAC 8, which in turn causes activation of HIF 1α and promotes angiogenesis [142]. Several compounds viz., TSA, vorinostat, FK228, butyrate and LAQ824, have been reported to inhibit the angiogenesis process and thereby decrease the expression of proangiogenic (HIF 1α and VEGF) factors. Several mechanisms are responsible for the HDACi-mediated degradation of HIF 1α. These include the degradation of HIF 1α by acetylation at Lys532 leading to ubiquitination, class II HDACs associate with HIF 1α and cause siRNA-mediated degradation of HIF 1α [143,144]. HDACi also degrades HIF 1α through compromising its transactivation potential, and also reduces the sensitivity of cancerous cells towards the angiogenic signal generated by VEGF [145,146]. Vorinostat and TSA downregulate the expression of VEGF receptors. Valproic acid impedes angiogenesis by increasing the production of thrombospondin-1 and activin A, the antiangiogenic proteins [147]. Use of combination therapies with VEGF inhibitors finds support from the above reports. 

### 7.5. ROS Generation

Oxidative stress has been implicated in cell death [148,149,150,151]. Experimental facts support the treatment of transformed cells with HDACi, such as TSA, vorinostat, butyrate or MS275, leading to the accumulation of reactive oxygen species (ROS) inside cells that subsequently induce cell death [152]. Ruelfi and co-workers [153] in 2001 reported that the quenching of ROS by N-acetylcysteine, a free radical scavenger decreases HDACi induced apoptosis, suggesting the role of HDACi in cell death. Thioredoxin (Trx), a hydrogen ion donor, is a ROS scavenger [154]. It is required in several redox reactions and is responsible for the activity of many protein, such as ribonucleotide reductase, which play an important role in the synthesis of DNA and the transcription factor. HDACi inhibits the activity of Trx by inducing the expression of Trx binding protein 2 (TBP2), which binds to it and inhibits its activity in cancer cells but not in normal cells [155]. Trx binds and inhibits the activity of ASK1. ASK1, an apoptosis signal regulating kinase, increases the rate of apoptosis by the activation of the SET1-JNK and MKK3/MKK6-p38 signaling pathways, and by elevating the expression of pro-apoptotic protein Bim. Therefore, higher expression of TBP2 by HDACi prevents the activity of Trx resulting in the increased expression of ASK1 and ultimately leading to apoptosis [156].

### 7.6. Mitotic Cell Death

HDACi treatment causes an abnormal acetylation pattern of histone protein in heterochromatin and centromere region. Newly synthesized chromatin contains acetylated histones and TSA treatment in transformed cells causes newly synthesized chromatin to remain acetylated, leading to the structural and functional disorder of centromere and pericentric heterochromatin [157], and this structural disturbance interferes with the phosphorylation of histone as well as disrupts the activity of mitotic spindle checkpoint proteins; i.e., BubR1, hBUB1, CENP-F, and CENP-E [123]. And this causes cell division arrest at the prometaphase stage followed by abnormal mitosis, such as the disaggregation and chromosomal loss leading to cell death either through apoptosis or mitotic cell death [2,124,158].

## 8. Potential Limitation and Side-Effects of HDACi

There are potential limitations to selective HDACi therapy. The effects of class I HDACi on DNA damage and repair pathways suggest that prolonged exposure to these agents could lead to unacceptable toxicities and secondary malignancies [159]. Class I HDACs may also play an oncogenic role depending on the context. Sontoro et al. [160] reported that in mouse tumor model, a single putative barrier to full transformation is surprisingly provided by HDAC 1 and HDAC2. Knock-down of HDAC 1 resulted in the both blockade of cellular differentiation and the increased genomic instability mediated by PML-RAR in hematopoietic progenitors. Either or both biological deregulations could be sufficient to cooperate with the tumor-promoting activities of oncoproteins, such as PML-RAR and Myc, and would provide a functional explanation for the observed increase in frequency of transformation to leukemia in HDAC1-deficient cells. Epigenetic changes are important for reprogramming somatic cells into pluripotent stem cells. Therefore, several inhibitors of epigenetic-modifying enzymes, including HDACi, are able to reprogram somatic cells into the pluripotent stem cells by modifying a chromatin structure and making it more permissive to transcription factors [119].

## 9. Conclusions

The present review summarizes the biological activities of HDAC. Further, emphasis has been given on the structural and functional features of the natural and synthetic inhibitors of HDAC as well as on their antitumor potential. HDACs are multisubstrate (histone and non-histone) enzyme involved in many biological processes; i.e., cell proliferation, differentiation, apoptosis, and senescence. According to scientific reports, histone deacetylase inhibitors have shown their efficacy as inhibitors of cancer initiation and progression. Generally, normal cells are resistant to HDACi, which selectively modulates gene expression in cancerous cells. Pan-HDAC inhibitors, such as vorinostat, belinostat, and panobinostat stimulate antitumor pathways, suggesting their therapeutic potential. FDA-approved HDACi are being used for the treatment of several types of cancers. Many HDACi are under clinical trial stages. HDACi show their anticancer action involving various mechanisms; i.e., cell cycle arrest, induction of apoptosis and autophagy in transformed cells, inhibition of angiogenesis, ROS as mediators of cell death, and mitotic cell death. Several natural HDACi are present in our diet: kaempferol (grapes, green tea, tomatoes, potatoes, and onions), resveratrol (grapes, red wine, blueberries and peanuts), sinapinic acid (wine and vinegar), diallyl disulfide (garlic) and zerumbone (ginger). Hence a better understanding of dietary HDACi regarding their target specificity and toxicity may underscore potential health benefits through nutritional intervention. Combination therapy might be another important direction to enhance the therapeutic efficacy of HDACi. Elucidation and validation of the detailed mechanistic aspects of HDACi action will provide a bright future for the use of HDACi as one of the important tools in the fight against cancer.

## Figures and Tables

**Table 1:** Histone deacetylase (HDAC) enzymes classification, number of amino acids, localization, function, protein targets and expression pattern.

Class	HDACs	Number of Amino Acid	Cellular Location	Chromosomal Location	Biological Function	Histone/Non-Histone Protein Target	Pattern of Expression of Gene
I	HDAC 1	483	Nucleus	1p35.2-p35.1	Proliferation and survival of cells	Histones, pRb, SHP, BRCA1, MECP2, ATM, MEF2, MyoD, p53, NF-κB, AR, DNMT1	Ubiquitous expression
	HDAC 2	488		6q21	Proliferation of cell and insulin resistance	Histones, BRCA1, NF-κB, MECP, GATA 2, pRb	
	HDAC 3	428		5q31.3	Proliferation and survival of cells	Histones, HDAC (4, 5, 7, 9), GATA 1, NF-κB, pRb	
	HDAC 8	377		Xq13.1	Proliferation of cell	HSP70	
IIA	HDAC 4	1084	Nucleus/Cytoplasm	2q37.3	Regulation of cytoskeleton dynamics and cell mobility	Histones, HDAC 3, 14-3-3, CaM, MEF 2	Tissue restricted expression
	HDAC 5	1122		17q21.31	Helps in endothelial cell function, gluconeogenesis, cardiac myocyte growth and function		
	HDAC 7	912		12q13.11	Helps in endothelial cell function and glyconeogenesis.		
	HDAC 9	1069		7p21.1	Helps in thymocyte differentiation, homologous recombination, cardiac cell function		
IIB	HDAC 6	1215	Cytoplasm	Xp11.23	Regulation of cytoskeleton dynamics and cell mobility	HDAC 11, SHP, HSP 90, α tubulin	Tissue restricted expression
	HDAC 10	669		2q13.33	Regulation of autophagy, homologous recombination.	LcoR, PP1	
III	SIRT 1	747	Nucleus/Cytoplasm	10q21.3	Autoimmunity, aging, redox balance, and cell survival	Histones, NF-κB, p53, p300	Variable expression
	SIRT 2	389	Nucleus	19q13.2	Survival, migration, and invasion of cell	Histone H4, PPAR-ϒ, p53, p300, α-tubulin, FOXO	
	SIRT 3	399	Mitochondria	11p15.5	Regulate ATP production and metabolism, cell signaling, apoptosis, urea cycle	Complex I of ETC, PGC-1α, p53, Ku70, Acetyl-CoA Synthetase, FOXO	
	SIRT 4	314		12q24.31	Energy metabolism, Urea cycle, cell signaling	Glutamate dehydrogenase	
	SIRT 5	310		6p23	Regulate ATP production and metabolism, cell signaling, apoptosis, urea cycle	Carbamoyl phosphate synthetase I, Cytochrome c	
	SIRT 6	355	Nucleus	19p13.3	Regulate metabolism	Histone H3, TNF-α	
	SIRT 7	400		17q25.3	Apoptosis	p53, RNA polymerase I	
IV	HDAC 11	347	Nucleus	3p25.1	DNA replication, Immunomodulation	HDAC 6	Ubiquitous in nature

AR, androgen receptor; ATM, ataxia-telangiectasia-mutated; BRCA, breast cancer; CaM, calmodulin; CoA, co-enzyme A; DNMT, DNA methyltransferase; FOXO, forkhead box O; GATA, GATA binding protein; HIF, hypoxia-inducible factor; HSP, heat shock protein; LcoR, ligand-dependent receptor co-repressor; MECP, methyl-CpG-binding domain protein; MEF, myocyte enhancer factor; NF-κB, nuclear factor-kappa B; PGC, peroxisome proliferator-activated receptor gamma coactivator; PP1, protein phosphatase; PPAR, peroxisome proliferator-activated receptor; pRb, retinoblastoma protein; SHP, Src homology region 2-domain-containing phosphatase; SIRT, sirtuin; TNF, tumor necrosis factor.

**Table 2:** Examples of the natural compound with histone deacetylase inhibitory activity.

S.N	Class of Compounds	Name of the Compound	HDAC Target	Source (Species/Family)	Structure	Reference
1.	Phenolics	Aceroside VIII	HDAC6	*Betula platyphylla*	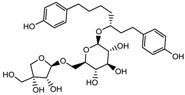	[87]
		Homobutein	Class I, II and IV	*Butea frondosa*	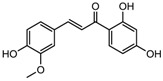	[88]
		Isoliquiritigenin	Class I, II and IV	*Glycyrhiza glabra*	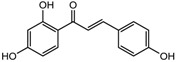	[89]
		Butein	Class I, II and IV	*Toxicodendron vernicifluum*	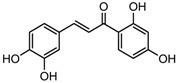	[88]
		Kaempferol	Class I, II and IV	*Aloe vera*	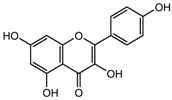	[89]
		Marein	Class I, II and IV	*Coreopsis maritima*	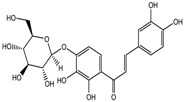	[88]
		Protocatechuic aldehyde	HDAC2	*Hordeum vulgare*	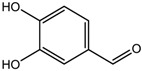	[23]
		Psammaplin A	Class I	*Poecillastra* spp. and *Jaspis* spp.	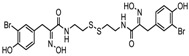	[23]
		Sinapinic acid	Pan-HDAC	*Hydnophytum formicarum Jack*	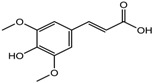	[90]
		Resveratrol	Class I, II and IV	*Vitis vinifera*	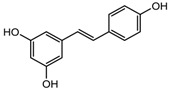	[91]
2.	Polyketides	Depudecin	HDAC 1	*Alternaria brassicicola*	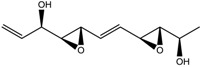	[92]
3.	Tetrapeptide	Apicidin	Class I HDAC	*Fusarium* spp.	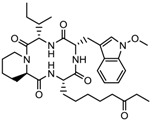	[93]
		Azumamide E	Class I	*Mycale izuensis*	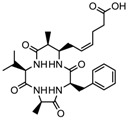	[94]
		Chlamydocin	HDAC 1, 6	*Diheterospora chlamydosporia*	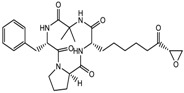	[95]
		Trapoxin A	Class I	*Helicoma ambiens* RF-1023	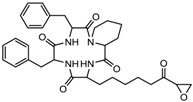	[96]
4.	Terpenoids	Zerumbone	Pan HDAC	*Zingiber zerumbet*	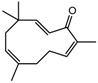	[23]
		β-Thujaplicin	HDAC 2	*Cupressaceae* spp.	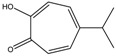	[23]
		6-methoxy-2*E*,9*E*-humuladien-8-one	Pan HDAC	*Zingiber zerumbet*	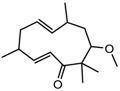	[23]
5.	Alkaloid	Lycorine	Pan HDAC	*Amaryllidaceae*	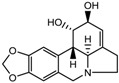	[97]
6.	Fatty acid	9-Hydroxystearic acid	Class I	Lipid peroxidation product	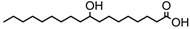	[98]
7.	Organosulphur compounds	Diallyl disulfide	Acetylation Level increased	*Allium sativum*	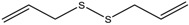	[99,100]
		(*S*)-allylmercaptocysteine		*Allium sativum*	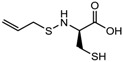	[101]
8.	Hydroxamic acid	Trichostatin A	Class I and II	*Streptomyces hygroscopicus*	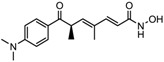	[2,11]
9.	Desipeptides	FK228	HDAC 1, 2	*Chromobacterium violaceum*	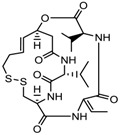	[11]
		Largazole	Class I	*Symploca* spp.	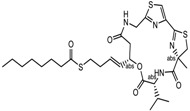	[99]

**Table 3:** FDA approved and under clinical trials histone deacytylase inhibitors (HDACi).

S.N	Chemical Class	Name of the Compounds	HDAC Target	Cancer Specificity	Trial Stage	Structure of the Compound	Reference
1.	Hydroxamic acid	SAHA (Vorinostat)	Class I, II and IV	CTCL	FDA approved (2006)	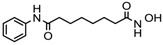	[107]
		Belinostat	Class I, II and IV	PTCL	FDA approved (2014)	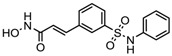	[108]
		Panobinostat	Class I, II and IV	MM	FDA approved in 2015	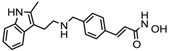	[109]
		Resminostat	Class I and II	Colorectal, HCC, HL	Phase II trial	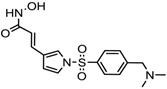	[6]
		Givinostat	Class I and II	CLL, HL, MM	Phase II trial	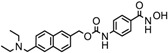	[110]
		Pracinostat	Classes I, II and IV	AML	Phase II trial	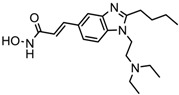	[111]
		Abexinostat	Class I and II	CLL, HL, Non-HL, Solid tumors	Phase I trial	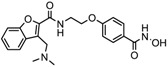	[45]
		Quisinostat	Class I and II	Solid tumor, CTCL	Phase I and II trial	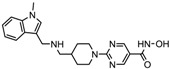	[45]
		MPT0E028	HDAC 1, 2 and 6	Solid tumor, B-cell lymphoma	Phase I trial	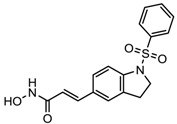	[112]
		CHR 3996	Class I	Solid tumors	Phase I trial	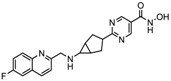	[113]
		CUDC 101	Class I and II	Solid tumor	Phase I trial	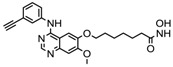	[6]
		CUDC 907	Class I and II	MM; lymphoma; solid tumor	Phase I trial	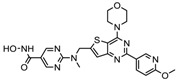	[6]
2.	Benzamides	Entinostat	Class I	Solid tumors	Phase I and II trial	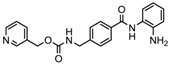	[113]
		Chidamide	HDAC 1, 2,3 and 10	Breast cancer	Phase II and III trial	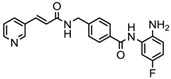	[114]
		Ricolinostat	HDAC 6	MM, Lymphoma	Phase I and II trial	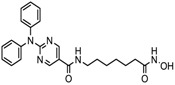	[115]
		Tacedinaline	Class I	Lung and pancreatic cancer, MM	Phase II and III trial	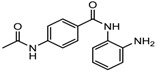	[6]
		Mocetinostat	Class I and IV	Solid malignancies	Phase I and II trial	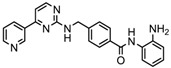	[116]
3.	Cyclic peptides	Romidepsin	Class I	CTCL, PTCL	FDA approved in 2009	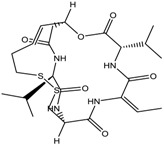	[6,117]
4.	Fatty acids	Valproic acid	Class I and II	Solid and hematological tumors	Phase I and II trial	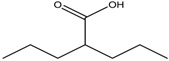	[118]
		AR-42	Class I and IIb	AML	Phase I trial	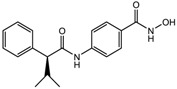	[45]
		Phenyl butyrate	Class I and II	Solid and hematological tumors	Phase I and II trial	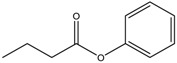	[118]
		Pivanex	Class I and II	NSCLC, Myeloma, CLL	Phase II trial	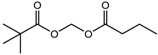	[113]

CTCL, cutaneous T cell lymphoma treatment; PTCL, peripheral T cell lymphoma; MM, multiple myeloma; HCC, hepatocellular Carcinoma; HL, Hodgkin lymphoma; CLL, chronic lymphocyte leukemia; AML, acute myeloid leukemia; NSCLC, non-small cell lung cancer.
